# CD137 Enhancement of HPV Positive Head and Neck Squamous Cell Carcinoma Tumor Clearance

**DOI:** 10.3390/vaccines2040841

**Published:** 2014-12-10

**Authors:** Christopher T. Lucido, Paola D. Vermeer, Bryant G. Wieking, Daniel W. Vermeer, John H. Lee

**Affiliations:** 1 Cancer Biology Research Center, Sanford Research, Sioux Falls, SD 57104, USA; E-Mails: ctlucido10@ole.augie.edu (C.T.L.); Paola.Vermeer@SanfordHealth.org (P.D.V.); Bryant.Wieking@coyotes.usd.edu (B.G.W.); Daniel.Vermeer@SanfordHealth.org (D.W.V.); 2 Sanford Health, 2301 East 60th Street North, Sioux Falls, SD 57104, USA

**Keywords:** CD137, immune, HPV, mouse, squamous

## Abstract

Standard-of-care cisplatin and radiation therapy (CRT) provides significant tumor control of human papillomavirus (HPV)-mediated head and neck squamous cell carcinomas (HNSCCs); this effectiveness depends on CRT-mediated activation of the patient’s own immune system. However, despite good survival, patients suffer significant morbidity necessitating on-going studies to define novel therapies that alleviate this burden. Given the role of the immune system in tumor clearance, immune modulation may further potentiate the CRT-activated response while potentially decreasing morbidity. CD137, an inducible cell surface receptor found on activated T cells, is involved in differentiation and survival signaling in T cells upon binding of its natural partner (CD137L). A number of studies have shown the effectiveness of targeting this immune-stimulatory pathway in regards to tumor clearance. Here, we test its role in HPV+ HNSCC tumor clearance using a previously characterized mouse model. We show that amplification of this stimulatory pathway synergizes with CRT for enhanced tumor clearance. Interestingly, tumor clearance is further potentiated by local tumor cell expression of CD137L.

## 1. Introduction

Human papillomavirus (HPV) infection defines a subclass of head and neck squamous cell carcinomas (HNSCCs). Despite presenting with more advanced disease, HPV positive (+) patients typically respond well to standard-of-care combined modality treatments which contributes to better outcomes as compared with their HPV negative (−) counterparts [1]. In fact, HPV status serves as a prognosticator of patient response to therapy and survival. While the incidence of HPV (−) HNSCC is declining, the prevalence of HPV (+) HNSCC cases is on the rise [2,3] making HPV (+) HNSCC patients a growing population. HNSCC patients suffer from significant morbidity; thus, research efforts aim to define new therapy combinations and/or novel drug targets to alleviate this burden. Using a murine HPV (+) HNSCC model, we have previously shown that HPV (+) tumors are curable in mice with combined cisplatin and radiation therapy (CRT), and that this clearance requires an immune response, mediated by CD4^+^ and CD8^+^ T cells [4,5].

Immune surveillance cells identify and target tumor cells for destruction [6,7]. Tumor growth and progression, therefore, may indicate an impaired immune response, or, alternatively, changes in the tumor cells themselves that allow for immune evasion [8,9]. While T cells identify a vast array of tumor antigens and consequently target and destroy tumor cells, some tumors escape through a wide variety of immune evasive mechanisms. Our understanding of how standard therapies provide a window of opportunity for altering tumor-mediated immune evasive strategies has significantly increased in recent years, making it an opportune time to develop novel therapies that further enhance immune mediated therapies [10,11]. In fact, immune checkpoint therapies, which augment the immune response, demonstrate promising results in a number of studies [11,12,13].

CD137 (also 4-1BB), a member of the tumor necrosis factor receptor superfamily (TNFR), is an inducible cell surface receptor expressed by T cells following activation. Its natural ligand, CD137L (also 4-1BBL), is present on activated antigen-presenting cells (APCs). Signaling via the CD137:CD137L pathway results in activation, differentiation, and preferential survival of CD8^+^ T cells [14,15]. Amplification of this pathway via either activating monoclonal anti-CD137 antibody treatment or CD137L over-expression promotes T cell-mediated tumor rejection [16,17,18]. Currently, a fully humanized activating monoclonal anti-CD137 antibody has completed phase I and II trials for its anti-tumor properties in the context of melanoma, renal cell carcinoma, and ovarian cancer [19]. Although this approach is promising, these agents require testing in the context for which they will be used. Some adverse reactions, such as inhibition of humoral immunity and severe autoimmune toxicity, have been noted with systemic administration of immune modifying antibodies [20,21]. Challenges with gene transfer of CD137L have also been noted, as the strategy has proven ineffective in several systems where the activating anti-CD137 antibody produced tumor regression [22].

The relative success of T cell-mediated tumor rejection via CD137:CD137L pathway activation prompted us to test its effectiveness in the context of HPV (+) HNSCC. Given that the increased sensitivity to CRT evident in HPV (+) HNSCC is mediated by an induced immune response, we hypothesized that further enhancing this immune response would increase tumor rejection. Furthermore, as several studies have shown the efficacy of tumor-localized modification of immune regulatory pathways [23,24,25,26], we also questioned whether local tumor expression of CD137L would further enhance clearance. To test this hypothesis, we examined the effects of CD137 activation therapies using our previously characterized mouse model of HPV (+) HNSCC, comparing the effects of systemic activation (via activating monoclonal anti-CD137 antibody treatment) *versus* a tumor-localized activation (via overexpression of CD137L on tumor cells). Here, we show that agonistic anti-CD137 antibody synergizes with cisplatin/radiation therapy to decrease tumor growth *in vivo*. In addition, over-expression of CD137L on tumor cells increases tumor clearance and survival in mice treated with cisplatin/radiation therapy emphasizing the importance of the tumor microenvironment in response to therapy.

## 2. Experimental Section

### 2.1. Cells Lines and Cells Culture Conditions

Mouse oropharyngeal epithelial cells (MOEs) stably expressing HPV16 E6 and E7 together with activated H-Ras and Luciferase (mEERL), were previously derived via retroviral transduction and maintained as previously described [27,28]. mEERL cells are routinely engrafted into mice and have been used as an acceptable animal model for HPV positive disease [13,29]. mEERL cells stably expressing mouse CD137 ligand (mCD137L) were maintained under the same culture conditions as the parental mEERLs, with the inclusion of the appropriate antibiotics.

### 2.2. Sub-Cloning of mCD137L

pORF-m41BBL v16 (InvivoGen, San Diego, CA, USA), encoding CD137L, was cloned into pcDNA3.1/Zeo (Invitrogen, Grand Island, NY, USA). Briefly, m41BBL (mCD137L) coding sequence was amplified by PCR using the following primers:

Forward primer: 5'-GCGATAGCGAAGCTTCTGAGATCACCGGTAGGAGG-3'.

Reverse primer: 5'-GCGATAGCGTCTAGACCCTGCTCAGACCCCATA-3'.

Following a pre-incubation at 95 °C for 5 min, cycling conditions were as follows: 95 °C for 30 s, 65 °C for 45 s, 72 °C for 2 min repeating for 30 cycles. Final annealing was 72 °C for 5 min, and held at 4 °C. The mCD137L insert and pcDNA3.1/Zeo vector were cut with *Hin*d III and *Xba* I (New England Biolabs, Ipswich, MA, USA) followed by ligation and transformation into DH5α competent cells (Invitrogen). Multiple colonies were analyzed and positive clones were identified through DNA sequencing (Eurofins MWG Operon, Huntsville, AL, USA).

### 2.3. Transfection of mCD137L into mEERLs

mEERL cells were transfected with the pcDNA3.1/Zeo mCD137L expression construct using Lipofectamine 2000 (Life Technologies, Grand Island, NY, USA) according to the manufacturer’s recommendations. 24 h post transfection, cells were placed under antibiotic selection using 500 µg/mL Zeocin (Invitrogen) with untransfected mEERLs serving as a control. Cells growing under selection were ring cloned and multiple clones were tested by western blot and immunofluorescence for mCD137L expression.

### 2.4. Western Blot Analysis

mEERL mCD137L clones were grown to 70% confluence, rinsed with 1 × PBS and harvested with lysis buffer (50 mM Tris HCl pH 7.5; 150 mM NaCl; 5 mM EDTA; 2 mM Na3VO4; 100 mM NaF; 10 mM NaPPi; 10% glycerol; 1% Triton; 1 × Halt Protease Inhibitors; 17.4 µg/µL PMSF). Membranes were pelleted by centrifugation (10,000 rpm at 4 °C) and soluble proteins harvested. BCA protein assay was performed according to manufacturer’s directions (Pierce, Logan, UT, USA) and equal amounts of total protein were separated by SDS-PAGE, transferred to PVDF-membranes (Immobilon-P, Millipore, Billerica, MA, USA), blocked with 5% BSA, and incubated with the manufacture-recommended concentrations of the primary antibodies: CD137L ((D-20), sc-11819, Santa Cruz Biotechnology, Santa Cruz, CA, USA), GAPDH (AM4300, Life Technologies, Grand Island, NY, USA). Following washes, PVDF membranes were incubated with the appropriate horse radish peroxidase (HRP) conjugated secondary antibody, followed by incubation with substrate (Luminata, Millipore) and developed with a charge-coupled device (CCD) camera imaging system (UVP).

### 2.5. Immunofluorescence Staining and Image Acquisition

Cells were seeded on collagen coated 8-well chamber slides and grown to 80% confluence. Cells were fixed with 4% paraformaldehyde (Electron Microscopy Sciences, Hatfield, PA, USA), permeabilized with 0.2% TritonX-100 (Thermo Scientific, Waltham, MA, USA), blocked in Superblock (Pierce), and incubated with antibody (1:100). Following washes with phosphate buffered saline (PBS), cells were incubated with Alexa Flour-conjugated secondary antibody (Invitrogen), washed, and coverslips mounted with Vectashield plus DaPi (Vector Labs, Burlingame, CA, USA). Cells were analyzed by confocal microscopy (Olympus FlouView 1000, Center Valley, PA, USA).

### 2.6. Mice

Male C57Bl/6 mice (The Jackson Laboratory, Bar Harbor, ME, USA) were maintained at the Sanford Research Laboratory Animal Research Facility (LARF) in accordance with USDA guidelines. Experiments were approved by the Sanford Research IACUC and performed within institutional guidelines. Briefly, using a 23-gauge needle, mEERL parental cells, and those over-expressing mCD137 (OE) or minimally expressing mCD137L (ME) were implanted subcutaneously in the right hind flank of mice (*n* = 10/group for each experiment). Ten to fourteen days post tumor implantation, mice were anesthetized with 87.5 mg/kg ketamine and 12.5 mg/kg xylazine, and the hind limb treated locally with 8 Gy X-ray radiation weekly for 3 weeks (RS2000 irradiator, RadSource Technologies, Suwanee, GA, USA). Cisplatin (CalBiochem, Temecula, CA, USA) was dissolved in bacteriostatic 0.9% sodium chloride (Hospira, Lake Forest, IL, USA) at 20 mg/m^2^ and administered intraperitoneally concurrent with radiation therapy. For experiments in which mice were treated with antibody, mice were segregated into groups: those receiving anti-CD137 (300 ug/treatment; BioXCell, West Lebanon, NH, USA) or control IgG (400 μg/treatment; BioXCell). Mice were intraperitoneally injected on days 7, 9, and 12 post-tumor implantation. Mice receiving CRT were treated weekly for three weeks beginning at one week post tumor implantation, a treatment schedule which has historically provided good tumor control [4,13]. Tumor growth was measured using previously established techniques [5]. Animals were euthanized when tumor size was >1.5 cm in any dimension. Mice were considered tumor free when no measurable tumor was detected for a consecutive period of 2 months. Survival graphs were plotted by standardizing each mouse to an endpoint tumor volume of 2000 mm^3^. Statistical analysis for the survival graphs was performed using the log-rank test. *p* value less than 0.05 was considered significant.

## 3. Results and Discussion

### 3.1. Activating Monoclonal Anti-CD137 Antibody Treatment Inhibits Tumor Growth in Conjunction with Chemotherapy/Radiation

**Figure 1:**
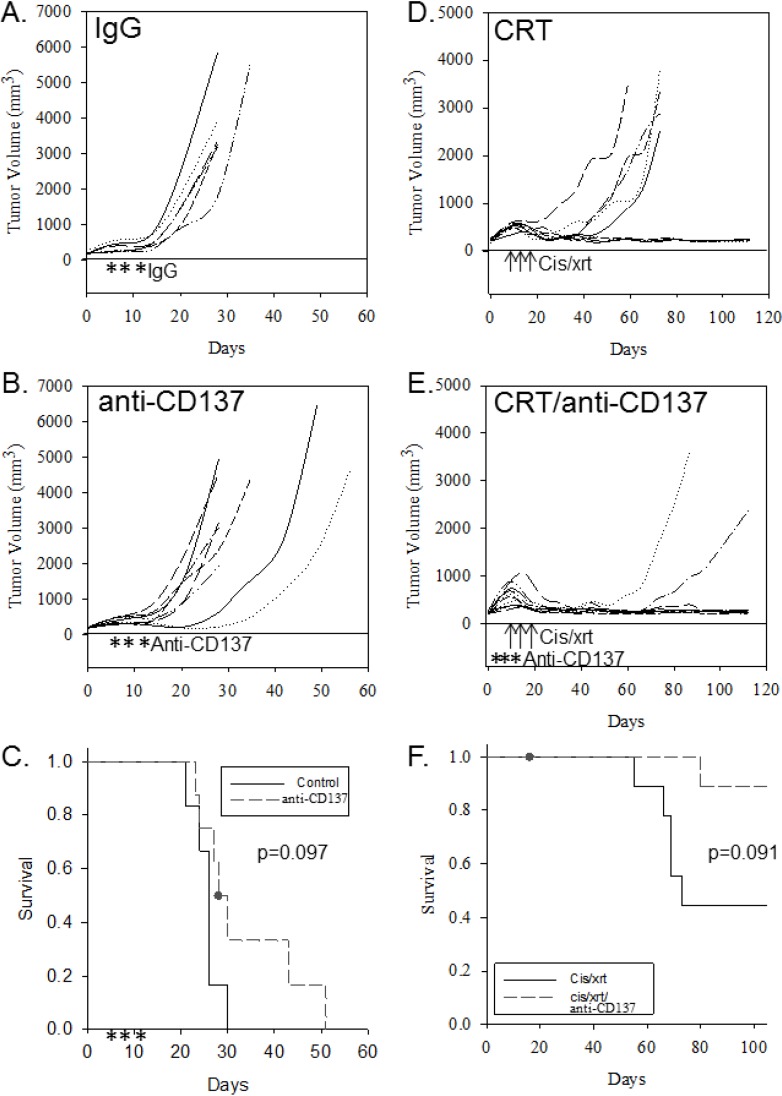
Activating anti-CD137 antibody administered in conjunction with standard-of-care treatment further inhibits tumor growth. Tumor growth curves (for individual mice) and group Kaplan-Meyer survival curves are shown for controls and for mice receiving treatment. Tumor growth curves for mice receiving (**A**) control IgG; (**B**) anti-CD137 antibody; (**D**) cisplatin/radiation (CRT) alone or (**E**) CRT in conjunction with anti-CD137 antibody. Kaplan-Meyer survival curves for (**C**) control mice and those treated with anti-CD137 antibody or (**F**) control mice receiving CRT or CRT together with anti-CD137 antibody. Arrows indicate three doses of radiation and cisplatin (8 Gy radiation and 20 mg/kg cisplatin on indicated days); asterisks indicate days of anti-CD137 antibody injections.

We have previously demonstrated that an immune response is required for clearance of HPV (+) cancers during treatment with CRT [4]. To determine the effects of an activating monoclonal anti-CD137 antibody in conjunction with standard therapy (cisplatin/radiation), mice were implanted with tumor and treated with activating anti-CD137 antibody alone or in combination with CRT. Mice treated with anti-CD137 antibody alone demonstrated no significant effect on tumor growth (Figure 1B) or survival (Figure 1C) as compared to no treatment/IgG controls (Figure 1A) (*p* = 0.097, Figure 1C). Mice treated with activating anti-CD137 antibody in conjunction with CRT demonstrated slowed tumor growth (Figure 1E) when compared with mice treated with CRT alone (Figure 1D). These findings suggest that the immune-mediated effects on tumor growth of HPV (+) tumors evident with CRT can be further enhanced using an immune stimulatory therapy, such as the activating monoclonal anti-CD137 antibody. However, this effect did not significantly improve survival (Figure 1F).

### 3.2. When Given with Cisplatin Alone or Radiation Alone, Anti-CD137 Antibody Synergizes Specifically with Cisplatin Treatment

**Figure 2:**
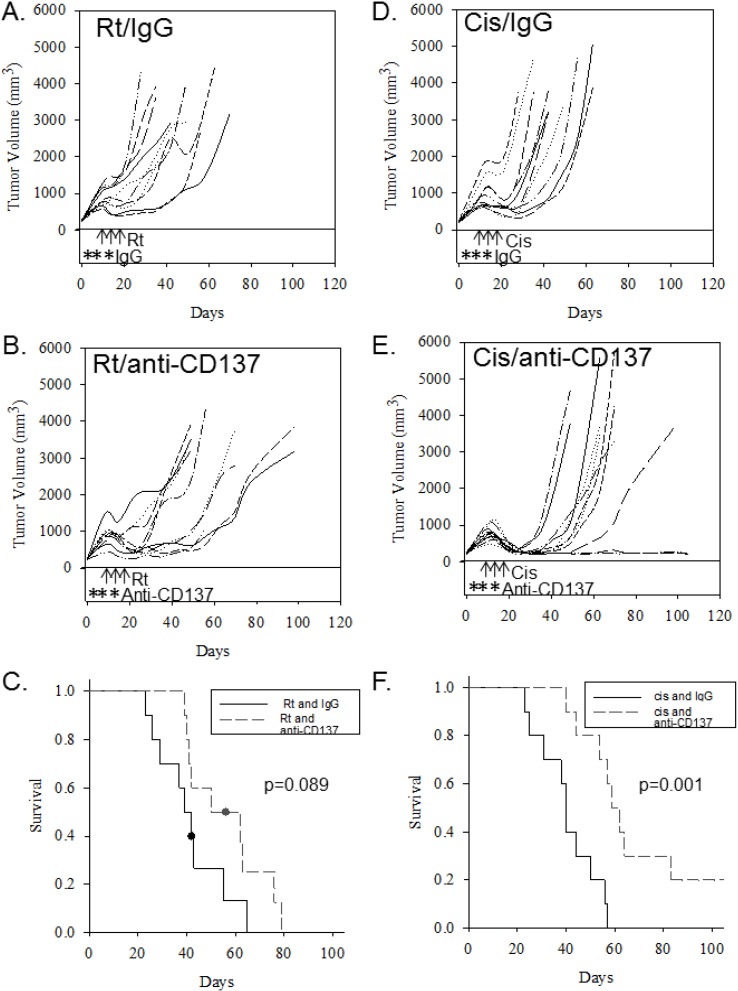
Administration of activating anti-CD137 antibody preferentially enhances the effects of cisplatin treatment. Tumor growth curves (for individual mice) and group Kaplan-Meyer survival curves are shown for mice receiving cisplatin or radiation with IgG, and for mice receiving cisplatin or radiation in conjunction with activating anti-CD137 antibody. (**A**) Growth curves of mice receiving three doses of radiation (8 Gy, on indicated days, arrows) and control IgG (on indicated days, asterisks); (**B**) Growth curves of mice receiving three doses of radiation (arrows) and anti-CD137 antibody (asterisks); (**C**) Kaplan-Meyer survival plot comparing mice in A and B; (**D**) Growth curves of mice receiving three doses of cisplatin (20 mg/kg, on indicated days, arrows) and control IgG (on indicated days, asterisks); **(E**) Growth curves of mice receiving cisplatin (arrows) together with anti-CD137 antibody (asterisks); (**F**) Kaplan-Meyer survival plots of groups in D and E.

To determine whether the activating monoclonal anti-CD137 antibody therapy synergizes with either cisplatin or radiation therapy, mice were implanted with mEERL cells and treated with anti-CD137 in combination with either cisplatin or radiation. While anti-CD137 augments antitumor effects of radiation therapy in murine breast and lung carcinoma models [30], our study found no significant improvement on survival (*p* = 0.089) when combined with radiation alone (Figure 2A–C). The combination of cisplatin and anti-CD137, however, resulted in a significant survival improvement compared to cisplatin alone (*p* = 0.001, Figure 2D–F), suggesting that addition of anti-CD137 antibody treatment to cisplatin therapy enhances the antitumor effects of cisplatin.

### 3.3. Over-Expression of CD137L on Tumor Cells Significantly Improves Antitumor Effects of Chemotherapy/Radiation

**Figure 3:**
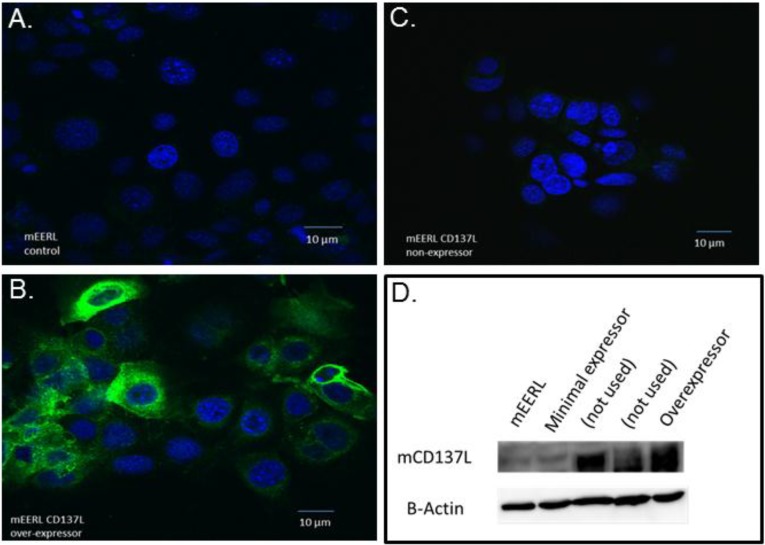
CD137L expression in parental mEERL cells and selected clones. *En Face* confocal images of mEERL cells and CD137L clones stained for CD137L (green) expression. Immunofluorescent analysis of CD137L expression in (**A**) parental mEERL cells; (**B**) a mEERL clone over-expressing CD137L; and (**C**) a mEERL clone minimally expressing CD137L. Nuclei are counterstained with DaPi (blue); (**D**) Western blot analysis of CD137L expression. Two additional clones are shown but were not used in these studies.

To determine whether tumor cells themselves activate the CD137 stimulatory pathway via CD137:CD137L interaction, we analyzed endogenous expression of CD137L on mEERL cells. Immunofluorescence and western blot data showed that mEERL cells lack CD137L expression (Figure 3A,D).

Some studies show that CD137L gene transfer elicits significant antitumor effects [31,32]. Others, however, have shown it to be ineffective in a number of systems where anti-CD137 antibody treatment has produced tumor regression [22]. Therefore, to test the role of tumor cell CD137L expression and its potential induction of the CD137:CD137L axis, murine CD137L was stably expressed in mEERL cells and individual clones evaluated for CD137L expression via immunofluorescence and Western blotting analysis (Figure 3B–D). One over-expressing (OE) clone was selected for *in vivo* analysis (Figure 3B,D). Another clone, growing under selection but with minimal CD137L expression (ME), was selected as an additional control (Figure 3C,D).

**Figure 4:**
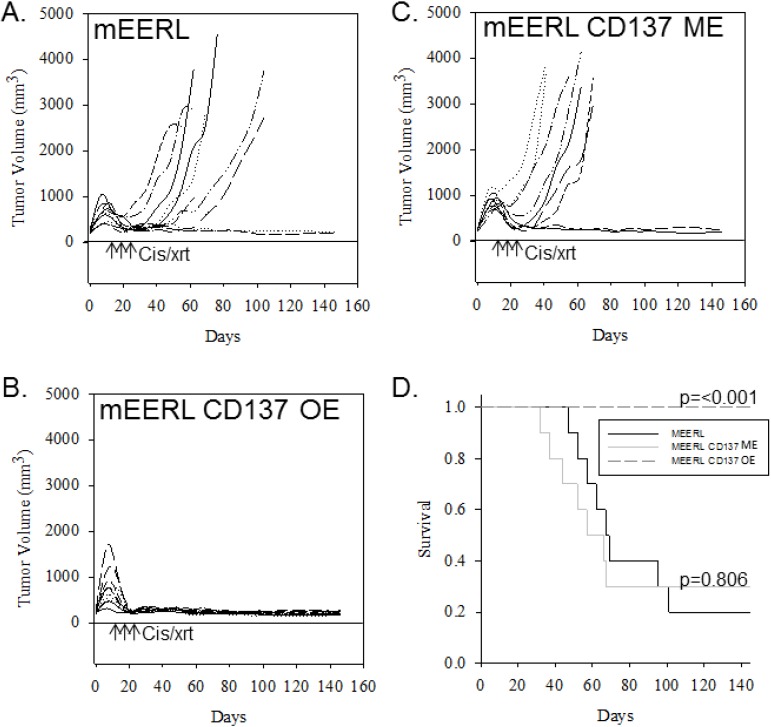
Over-expression of CD137L on tumor cells enhances anti-tumor effects of CRT. Tumor growth curves (for individual mice) and group Kaplan-Meier survival curves are shown for mice implanted with (**A**) parental mEERLs, (**B**) mEERLs over-expressing CD137L (OE), or (**C**) mEERLs minimally expressing CD137L (ME). Mice received three doses of cisplatin (20 mg/kg) and radiation (8 Gy) on indicated days (arrows), (**D**) Kaplan-Meier survival curves for mice in A, B and C.

To investigate the effects of tumor CD137L expression on standard-of-care treatment, we implanted these mEERL CD137L clones into mice. We found no statistically significant effect of CD137L expression on tumor growth or survival when mice were not treated with CRT (data not shown). However, treatment with CRT demonstrated a statistically significant improvement (*p* < 0.001) of survival (Figure 4D) and dramatic inhibition of tumor growth in mice with CD137L overexpressing tumors (Figure 4B). Importantly, all mice in this group cleared tumor (Figure 4B). Mice implanted with the CD137 minimally expressing clone responded similarly to CRT as the parental (mEERL) cells (Figure 4C). These findings demonstrate that tumor-localized expression of CD137L together with CRT may not only significantly inhibit tumor growth, but also improve long-term survival.

## 4. Discussion

The antitumor effects of immune-modulating therapies have shown promise for various cancers. Therefore, we questioned whether activation of the CD137:CD137L co-stimulatory pathway would enhance immune clearance of HPV (+) cancers either as a single agent, in combination with CRT, and when expressed locally by the tumor cells themselves (*i.e.*, not administered systemically). In this study, we show that agonistic CD137 antibody treatment inhibits tumor growth when given in conjunction with standard CRT treatment in a murine model of HPV+ HNSCC. We demonstrate that this effect is likely mediated by synergy between cisplatin (rather than radiation) and the anti-CD137 antibody.

Multiple co-stimulatory signals affect immune recognition and activation. Thus, our finding that neither activating monoclonal anti-CD137 antibody treatment nor over-expression of CD137L on tumor cells alone was sufficient to affect tumor clearance is not surprising. In fact, modulating other costimulatory pathways has led to significant clinical success in other pre-clinical models [33] as well as in humans [34]. However, our observation that these strategies improve anti-tumor activity in conjunction with standard CRT suggests a possible route to further augment the immune response activated in HPV (+) HNSCC during CRT. Previous studies have demonstrated the anti-tumor effects of anti-CD137 antibody treatment in combination with radiation [30], however, until now, none have demonstrated its effects in conjunction with cisplatin and radiation in the context of HPV (+) HNSCC. Studies have increasingly recognized the role of HPV in causing cervical, anal, and oropharyngeal tumors. Cisplatin and radiation therapy is commonly used in the treatment of many of these cancers. This study shows the promise of CD137:CD137L signal amplification in stimulating the immune response activated during standard treatment, which is required for HPV (+) tumor clearance. Our findings suggest that further investigation of immune modulatory therapies, in conjunction with standard treatment, could prove efficacious in the context of HPV (+) cancers. These data suggest that strategies of tumor-directed CD137:CD137L amplification should be investigated further as a means to enhance tumor directed immune activity.

## 5. Conclusions

We demonstrate that amplification of the CD137:CD137L pathway synergizes with CRT enhancing tumor clearance in HPV+ HNSCC. Tumor clearance is further potentiated by local tumor cell expression of CD137L. These finding suggest that immune modulatory therapies together with standard treatment can significantly improve outcomes for HPV + cancers and should be further investigated.
